# Uncoupling genotoxic stress responses from circadian control increases susceptibility to mammary carcinogenesis

**DOI:** 10.18632/oncotarget.15678

**Published:** 2017-02-24

**Authors:** Mingzhu Fang, Pamela A. Ohman Strickland, Hwan-Goo Kang, Helmut Zarbl

**Affiliations:** ^1^ School of Public Health, Rutgers, The State University of New Jersey, Piscataway, NJ, USA; ^2^ Environmental and Occupational Health Sciences Institute, Rutgers, The State University of New Jersey, Piscataway, NJ, USA; ^3^ NIEHS Center for Environmental Exposures and Disease, Rutgers, The State University of New Jersey, Piscataway, NJ, USA; ^4^ Cancer Institute of New Jersey, Rutgers, The State University of New Jersey, Piscataway, NJ, USA; ^5^ Veterinary Drugs & Biologics Division, Animal and Plant Quarantine Agency, Gimcheon-si, Gyeongsangbuk-do, Republic of Korea

**Keywords:** genetic susceptibility, circadian rhythm, DNA damage response and repair, Period 2, SIRT1

## Abstract

We previously demonstrated that chemopreventive methylselenocysteine (MSC) prevents *N*-Nitroso-*N*-methylurea (NMU)-induced mammary carcinogenesis in the susceptible Fischer 344 (F344) rats by enhancing NAD^+^-dependent SIRT1 activity, restoring circadian expression of *Period 2 (Per2)* and circadian controlled genes. Here, we show that compared to the genetically resistant Copenhagen (COP) rat strain, mammary glands of the F344 rats have a 4-hour phase delay in circadian expression of *Per2*. Consequently, F344 rats failed to increase SIRT1 activity and circadian expression of *Per2* and DDRR genes after exposure to NMU. Exposure of COP rats to NMU had the opposite effect, enhancing SIRT1 activity, increasing circadian expression of *Per2* and DDRR genes. Significantly, SIRT1 activity and circadian expression of *Per2* and DDRR genes in NMU-treated F344 rats on a chemopreventive regimen of MSC approximated those in NMU-treated COP rats. These results indicated that COP rats have an increased capacity to maintain NAD^+^-dependent SIRT1 activity under genotoxic stress. This contention was supported by increased stability of the period and phase of circadian locomotor activity in COP vs F344 rats exposed to changing light conditions. The increased sensitivity and rapid response of COP to changing light were correlated with the enhanced circadian response of this strain to carcinogen. Disturbance of circadian rhythm by jet lag also disrupted circadian expression of *Per2* and DDRR genes, and accelerated mammary tumorigenesis in rodent models. These results suggested that uncoupling of DDRR responses from circadian control by environmental stresses and endogenous factors increases susceptibility to mammary carcinogenesis, possibly by inducing a promutagenic state.

## INTRODUCTION

The circadian clock regulates biological processes ranging from gene expression to sleep behavior in a precise and sustained rhythm with a periodicity of ~24 hours. This molecular oscillator functions in the central pacemaker (suprachiasmatic nucleus), SCN and cells comprising most peripheral tissues. In mammalian cells, periodicity of the circadian clock is regulated by interconnected transcriptional/translational feedback loops. Heterodimers of BMAL1 and either CLOCK or NPAS2 regulate transcription by binding to E-box elements in the promoters of core circadian genes (CGs) including *Per*, *Cry*, *ROR*, and *Rev-ErbAα*. The CGs, in turn, regulate the expression of circadian-controlled genes (CCGs), including hormone receptors, growth-regulatory genes and DNA damage response and repair (DDRR) genes [1]. As they accumulate in the cytoplasm, heterodimers of PER:CRY are post-translationally modified and transported to the nucleus, where they repress CLOCK:BMAL1 transcriptional activity. In this way, core circadian genes limit their own transcription and set up the rhythmic expression of CGs and CCGs [2]. The histone acetyl transferase activity of the CLOCK transcription factor plays a critical role in circadian gene expression. CLOCK acetylates its binding partner BMAL1, as well as histone 3 associated with circadian gene promoters [3]. NAD^+^-dependent SIRT1 protein deacetylase counterbalances CLOCK-directed acetylation. Regulation of SIRT1 by NAD^+^/NADH levels links cellular metabolic pathways to circadian regulation [4]. These intrinsic molecular oscillators are synchronized and can be reset by external signals including light, genotoxic stress, nutrients, hormones, and environmental signals [1, 2, 5, 6]. In this way, circadian clocks integrate a wide variety of endogenous and exogenous inputs to maintain normal cellular and physiological homeostasis under changing environmental conditions.

Disruption of circadian rhythm by lifestyle, occupational, environmental, and genetic factors increases risk of various chronic diseases in humans and other mammals [7, 8]. Epidemiological studies indicate that chronic shift work is associated with increased risk of cancer, especially breast and prostate cancer. Animal studies have shown that circadian disruption resulting from SCN ablation or chronic jet lag accelerated growth of transplanted tumors in mice up to three-fold [9, 10]. Constant light or chronic jet lag promote carcinogen-induced mammary tumorigenesis and liver carcinogenesis in rodents [11, 12]. However, neither SCN ablation, nor chronic jet lag increased corticosterone secretion, a “classical” response to stressors, suggesting a prominent role for circadian disruption in carcinogenesis. Circadian disturbances were also found in up to 50% of patients with metastatic cancer and are associated with poor outcomes [13]. Subsequent mechanistic studies indicated that the circadian clock regulates transcription, translation, and/or post-translational modification of ~10% of genes and/or proteins in mammalian cells, including most genes involved in DDRR signaling pathways and cell-cycle control [14]. Genotoxic agents can reset circadian rhythm through interlocking regulation of CGs (e.g., Per2) and ATM/Chk2 in P53-dependent manner, leading to protection from DNA damaging processes and carcinogenesis [15–19]. Per2 also links the circadian cycle to estrogen receptor signaling [20, 21]. Knocking out or mutating CGs (e.g., *Per2*) therefore increases cancer cell growth, and accelerates spontaneous and carcinogen-induced tumor development in rodents [22, 23]. By contrast, normal or ectopic expression of clock genes induces cell cycle arrest and sensitizes cancer cells to DNA damage-induced apoptosis [24, 25]. Mutation of DDRR genes (e.g., ATM) disrupts circadian control and/or prevents resetting of the clock in response to genotoxic stress [15, 26]. Down-regulation of CGs is observed in many human cancers, and genetic variants, mutations, and epigenetic modifications of CGs are associated with invasive breast cancer [27–36]. In the present study, we investigated the effects of uncoupling DDRR processes from circadian control on sensitivity to genotoxicants and genetic susceptibility to mammary cancer.

Rat strains show tremendous genetic variation in susceptibility to mammary carcinogenesis. Pubescent females of most laboratory strains, including Fisher 344 (F344) rats, are susceptible to mammary carcinogenesis when exposed to chemicals, hormones, or radiation. By contrast, feral rats and the Copenhagen (COP) and Wistar-Kyoto strains are highly resistant [37]. A single carcinogenic dose of NMU induces mammary carcinogenesis in female rats from different strains including Wistar [38], F344 [39], Lewis [40] and Sprague Dawley [41, 42], and circadian rhythms have been studied in all these strains [39, 43–45]. Susceptible strains vary in their sensitivities to chemicals, hormones and radiation, and are known to have differences in the circadian rhythms of circulating hormones and inflammatory cells [37, 45]. F344 female rats treated with a single carcinogenic *i.p*. dose (50 mg/kg body weight) of NMU develop mammary tumors, beginning at ~10 weeks after exposure. Tumor incidence increases to ~30% by 24 weeks and peaks at ~83% at 32 weeks post-exposure [46]. By contrast, COP rats are highly resistant to *N*-nitroso-*N*-methylurea (NMU)-induced mammary carcinogenesis, with a tumor incidence of 0% by 24 weeks. Only ~5% of pubescent COP females treated with the prolactin releasing drug, perphenazine, and NMU developed mammary adenocarcinomas with latencies comparable to F344 strain [47]. Sensitivity to carcinogenesis in rats is a polygenic trait, with more than 30 *mammary carcinoma susceptibility (Mcs)* loci identified to date [48, 49]. Our studies using a cross between the F344 and COP strains identified *Fry* as a *Mcs* gene, whose expression is significantly associated with estrogen receptors status [50] [20, 21]. Our previous studies also found that prolactin and its cell surface receptor (PrlR) were elevated in susceptible strains and contributed to mammary tumorigenesis [47]. These findings indicated that the molecular mechanisms underlying susceptibility to mammary carcinogenesis include differential regulation of estrogen and prolactin pathways, both of which are under circadian control [51–54].

Our more recent studies demonstrated that exposure to NMU disrupts circadian expression of most CGs, including *Per2* and CCGs involved in DDRR and cell proliferation, during early stage of tumorigenesis. As in other strains treated with carcinogens [55, 56], chemopreventive regimens of dietary methylselenocysteine (MSC) reduced mammary tumor incidence in F344 rats by 63% [46]. MSC also restored and enhanced circadian gene expression in NMU-treated this strain [20, 46]. We demonstrated that both NMU and MSC mediated their effects by modulating the epigenetic regulation of circadian gene expression through the NAD^+^-dependent SIRT1 pathway [57]. In the present study, we compared circadian physiology (including locomotor activity and circulating hormones), circadian gene expression, and the underlying epigenetic mechanisms in mammary glands of differentially susceptible rat strains at baseline, and after exposure to carcinogens (jet lag or NMU). We also determined the effects of chemopreventive MSC on these endpoints. Finally, we determined the promoting effects of circadian disruption by jet lag in a transgenic mouse model of metastatic breast cancer.

## RESULTS

### Differential baseline expression of core CGs in F344 vs COP strains

**Figure 1:**
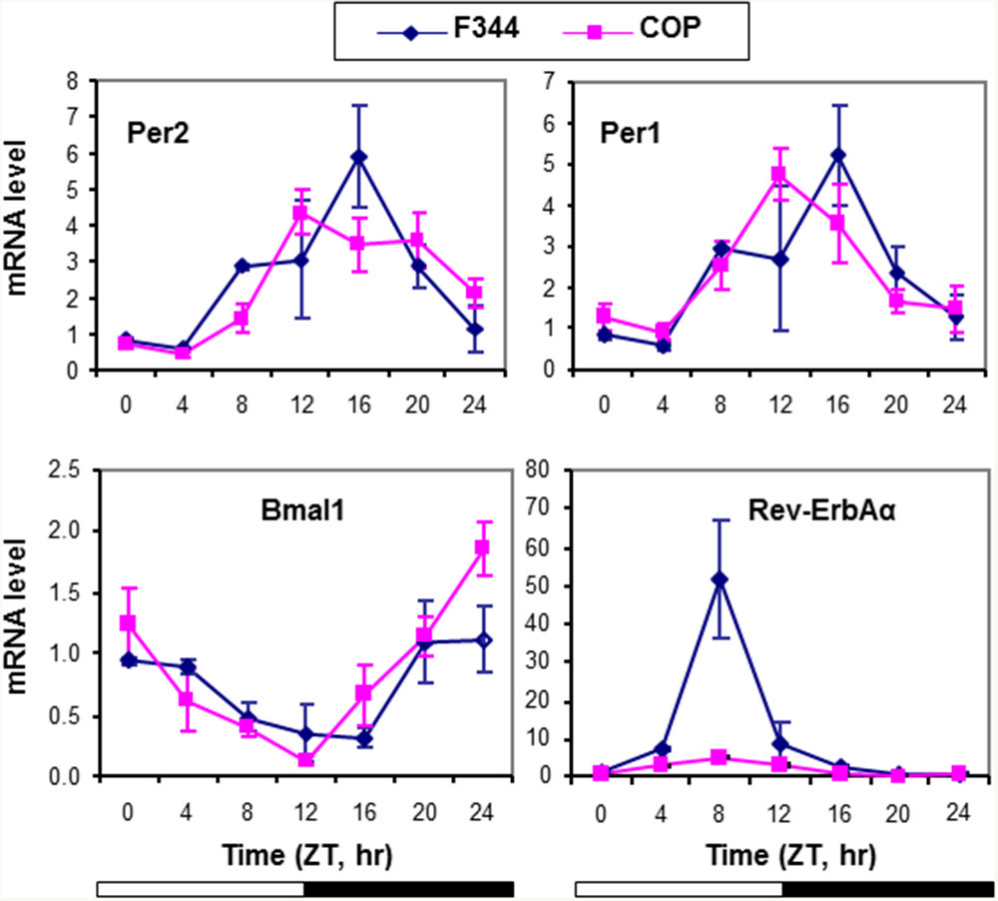
Circadian mRNA expression of core CGs in mammary glands of F344 and COP rats Three pubescent female COP (square) or F344 (diamond) rats were sacrificed every 4 hours over a 24 hour period, beginning at 7 AM. Relative mRNA expression levels were determined with RT-qPCR. X-axis: Zeitgeber time (lights-on at ZT0, 6 AM and lights-off at ZT12, 6 PM); empty bar indicates lights-on and solid bar indicates lights-off. Y-axis: relative mRNA levels.

To investigate the association between altered circadian control and differential genetic susceptibility to mammary cancer, we compared the expression of four core CGs in mammary glands of pubescent female F344 and COP rat strains over a 24 hour period. Relative to COP rats, F344 rats showed a 4 hour delay in their circadian phase relative to lights being turned off at Zeitgeber Time 12 (ZT12). Transcription of *Per1* and *Per2* peaked at ZT16 in F344 mammary glands. Expression of these core circadian genes in the COP rats was maximal at ZT12, right after lights-off (Figure 1). Compared to COP rats, F344 rats also showed elevated circadian expression of *Rev-ErbAα* mRNA (Figure 1), a core circadian gene involved in adipogenesis [58]. These results indicated significant strain differences in baseline regulation of circadian control.

### Strain specific differences in circadian regulation of DDRR genes

**Figure 2:**
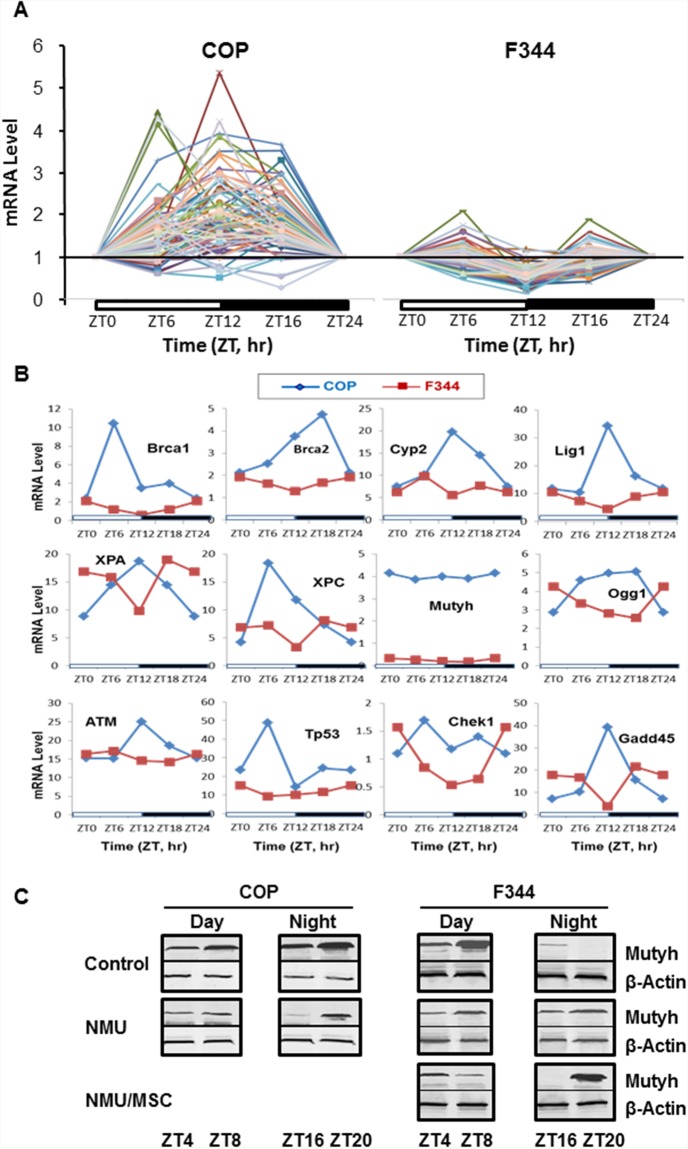
Circadian mRNA expression of DDRR genes in mammary glands of F344 and COP rats Three rats per group were sacrificed every 4 or 6 hours over a 24 hour period, beginning at 7 AM. **(A)** mRNA levels of 84 DDRR genes were determined in pooled total RNA samples from 3 rats per time point per group using RT-qPCR arrays (Qiagen). The expression level of each gene was normalized with that at ZT0. **(B)** Comparison of mRNA levels of representative genes in A without normalization with the level at ZT0. X-axis: Time (ZT, hour); Y-axis: relative mRNA levels.; **(C)** Protein levels of Mutyh were determined with Western Blot (WB) in total protein samples pooled from 3 rats per time point per group. Representative WB images for Mutyh protein levels during day or night were presented. B-Actin was used as loading control.

We and others previously showed that circadian rhythm controls the expression of many genes involved in DDRR. To evaluate the effects of strain differences in circadian regulation on expression of DDRR genes, we compared mRNA levels for a panel of 84 genes (Supplementary Table 1) using Rat DNA Damage Signaling Pathway RT-qPCR Arrays. In COP mammary glands, 45% of DDRR genes showed increased circadian expression over a 24 hour period, with peaks at ZT12. Moreover, 76 out of 84 genes showed increased circadian expression relative to the levels in F344 rats. By contrast, 30% of these genes showed decreased expression at ZT12 in F344 rats (Figure 2A & 2B). Mutyh, which repairs 8-oxo-guanine adducts, had dramatic differences in mRNA expression level (F344>COP), although expression not show a circadian pattern of transcription in either strain (Figure 2B). However, circadian expression of Mutyh was evident at the protein level. While the F344 showed higher levels of Mutyh during the day, the COP strain had higher protein levels at night (Figure 2C). These diametrically opposed patterns in DDRR gene expression suggest that differences in DNA repair capacity may contribute to the differential susceptibility of these two strains to mammary carcinogenesis. We therefore compared the expression of DDRR genes in mammary cells after exposure to a carcinogenic dose of NMU.

### Strain specific differences in circadian response to genotoxic stress

**Figure 3:**
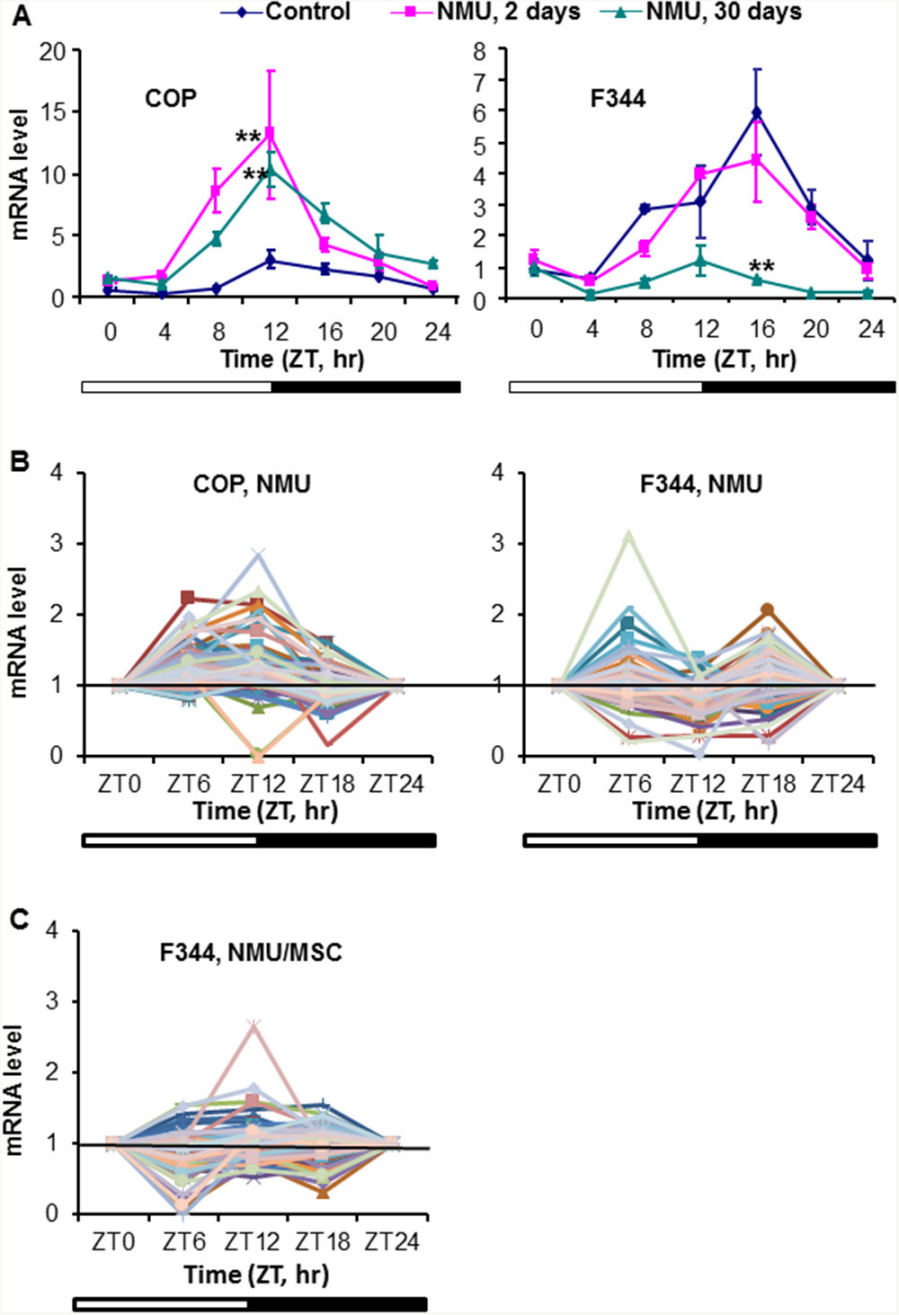
Differential response of *Per2* and DDRR gene expression to NMU in mammary glands of F344 and COP rats **(A)** Three of pubescent female F344 or COP rats per group were sacrificed every 4 hour over a 24 hour period, beginning at 6 AM, at days 0 (diamond), 2 (square), or 30 (triangle) post-exposure to NMU. mRNA expression levels were determined with RT-qPCR. The Per2 mRNA expression in F344 rat (right) was previously reported and is licensed under CC BY 2.0. **(B)** Three pubescent female F344 or COP rats per group were sacrificed every 6 hours over a 24 hour period, beginning at 6 AM, at day 30 post-exposure to NMU. mRNA levels of 84 DDRR genes were determined with RT-qPCR array in total RNA samples pooled from 3 rats per time point per group. **(C)** DDRR mRNA expression in mammary glands of F344 rats maintained on MSC diet for 30 days following exposure to NMU. The expression level of each gene was normalized with that at ZT0. **Indicates statistically significant difference at p<0.01 compared to control group (one way ANOVA, n=3). X-axis: Zeitgeber time (ZT); Y-axis: relative mRNA level, mean ± SE (n=3).

To compare strain differences in circadian responses to genotoxic stress as a function of time, we compared expression of *Per2* mRNA in mammary glands of COP and F344 rats at days 2 and 30 after exposure to NMU. As reported in our previous publication, we demonstrated a slight reduction in circadian expression of *Per2* mRNA at day 2 after exposure to a single carcinogenic dose of NMU, progressing to complete ablation at day 30 in mammary glands of the susceptible F344 rats (Figure 3A-right, cited from reference [57]). In stark contrast to decreased circadian expression observed in the susceptible F344 rats, NMU actually increased rhythmic expression of *Per2* gene in mammary glands of COP rats at day 2 after exposure. Moreover, the increased circadian expression was sustained for at least 30 days post-exposure (Figure 3A-left).

We next examined how strain differences in circadian responses to genotoxic stress affect levels and rhythmicity of DDRR genes expression. Since rhythmic expression of *Per2* in F344 rats was abolished 30 days after exposure to NMU, the expression of DDRR genes in mammary glands of exposed F344 rats should be low and lack circadian rhythmicity. As expected, NMU exposure did not significantly change the circadian expression patterns of DDRR genes in mammary glands of F344 rats. By contrast, mammary glands of COP rats maintained a circadian pattern of DDRR gene expression, with most peak levels at night (ZT12). Circadian expression was maintained for at least 30 days following exposure to NMU, although the amplitudes were reduced compared to control COP rats (Figure 3B). Significantly, chemopreventive MSC restored the circadian mRNA expression of many DDRR genes in NMU-treated F344 rats towards those seen in NMU-treated COP rats (Figure 3C). MSC also restored the circadian pattern of Mutyh protein in NMU-treated F344 rats towards levels and pattern (night>day) in COP rats (Figure 2C). These findings suggest that the genetic block to mammary carcinogenesis in the COP strain results in part from the ability to maintain the circadian expression of DDRR genes in the presence of genotoxic insult.

### Strain specific differences in SIRT1 activity following genotoxic insult

**Figure 4:**
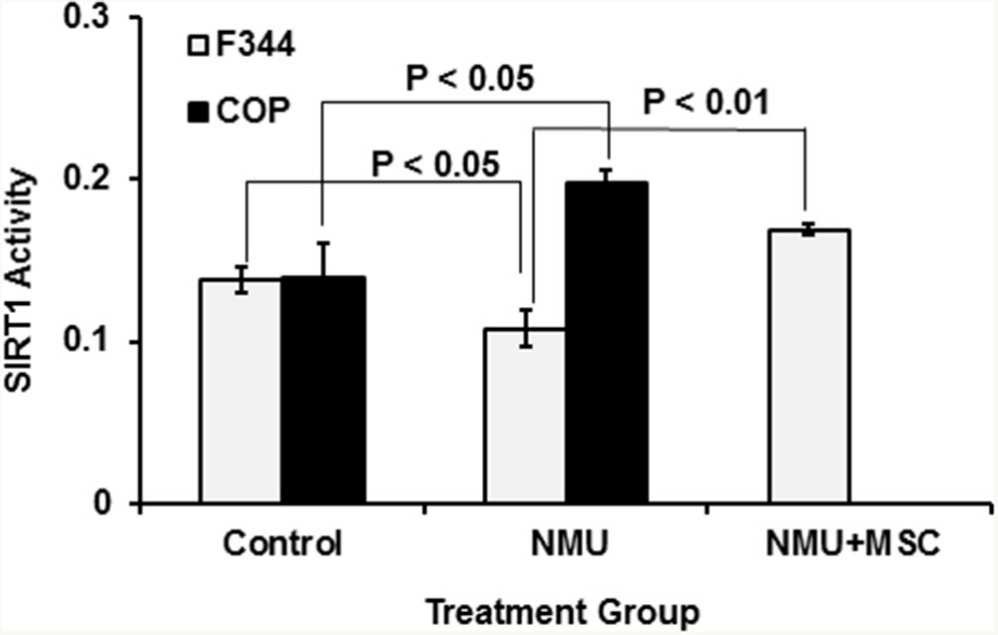
Comparison of SIRT1 activity in mammary glands of COP vs F344 rats after exposure to NMU Both F344 and COP rats (3 per group) were exposed to NMU or saline (control) and maintained on control diet, while an additional three NMU-treated F344 rats were maintained on an MSC-enriched diet (3 ppm Se). After 30 days, animals were sacrificed at ZT12 (after lights-off at 6 PM) and total protein was extracted from harvested mammary glands. Protein deacetylase activity was determined with the SIRT1 Fluorimetric Drug Discovery Kit. Enzyme activities were normalized to protein concentration. Significance was evaluated and presented at p<0.05 or p<0.01(one-way ANOVA, n=3).

Previous studies showed that NAD^+^-dependent SIRT1 protein deacetylase activity regulates circadian rhythm by altering acetylation of BMAL1 and histones on circadian gene promoter regions [59]. We demonstrated that NMU altered the acetylation of BMAL1 and histone 3 on the *Per2* promoter by inhibiting NAD^+^-dependent SIRT1 activity [57]. Moreover, a chemopreventive dose of MSC restored SIRT1 activity in mammary cells *in vivo* and *in vitro*. We therefore compared SIRT1 activity in mammary glands of COP and F344 rats fed a control or a MSC-enriched diet for 30 days after exposure to vehicle or a single carcinogenic dose of NMU. While exposure to single carcinogenic dose of NMU increased SIRT1 activity in mammary glands of COP rats, it decreased SIRT1 activity in F344 rats (Figure 4). Moreover, alteration of SIRT1 activity in mammary glands was temporally correlated to changes in rhythmic expression of *Per2* (Figure 3A). Significantly, MSC restored SIRT1 activity in NMU-treated F344 rats towards levels seen in the COP rats (Figure 4). These observations suggested that SIRT1-mediated differential acetylation of BMAL1 and histones contributes to the differential circadian responses of mammary cells of these two rat strains to NMU.

### Genetic regulation of circadian locomotor activity and hormone profile

**Figure 5:**
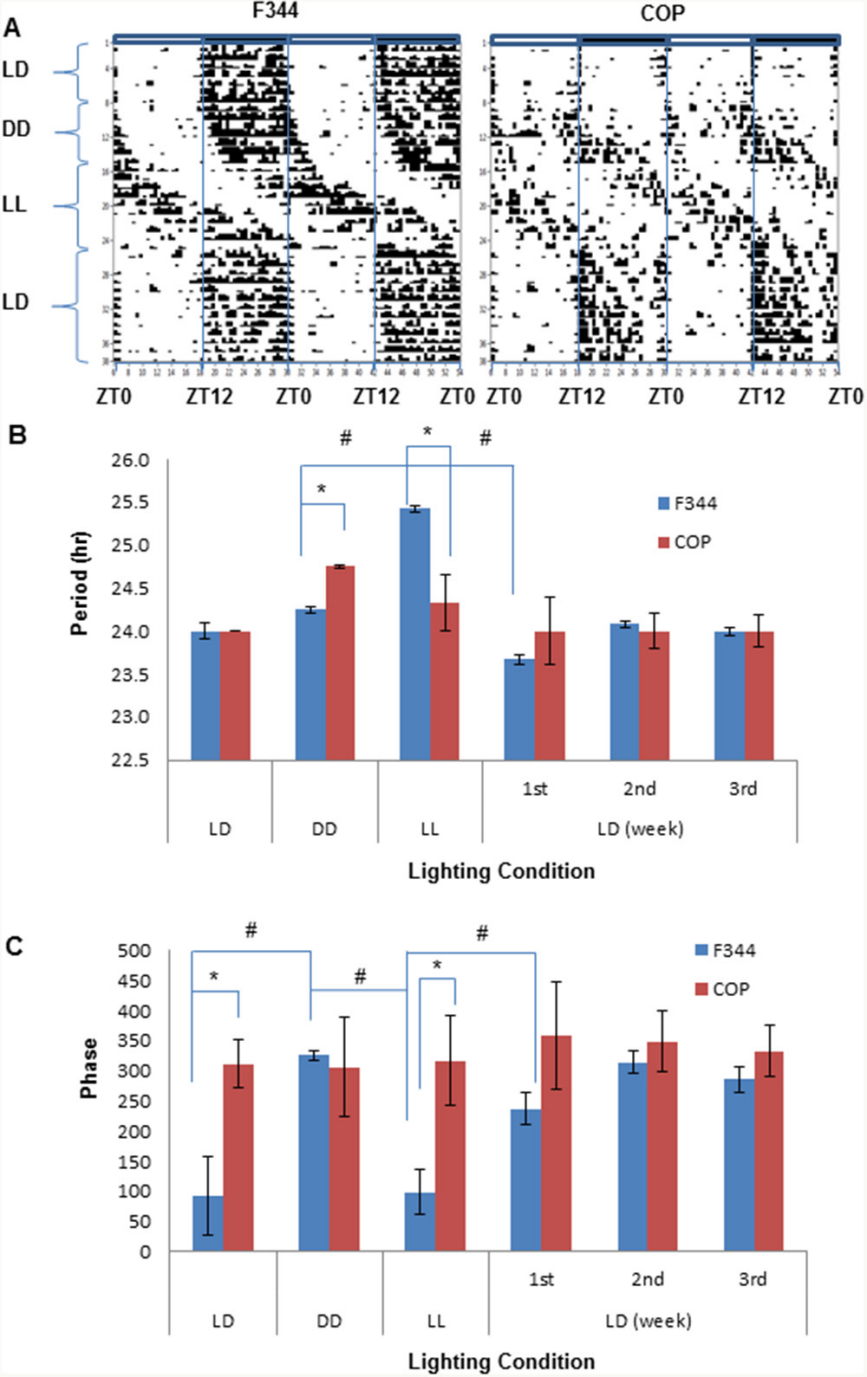
Comparison of locomotor activities between COP and F344 rat strains Free wheel running activity was recorded with ClockLab for 10 weeks under different lighting conditions, including 2-week regular 12 hour LD cycles, 2-week DD, 19 day LL, before being returned to 23 days regular LD cycles. **(A)** Representative actograms. Activity counts are indicated by the vertical black marks in the activity record. The records are double plotted so that each horizontal line presents two days from the lights on at 6 am (Zeitgeber time 0, ZT0); **(B)** Period; **(C)** Phase. Values of mean ±SE (n=3 for each) were presented. * and ^#^ indicate intergroup and intragroup statistical significance at P<0.05, respectively, in **(B)** and **(C)**.

We also compared endogenous circadian rhythms and their responses to light change by monitoring locomotor activity in the F344 and COP rats. Activity was continuously monitored and analyzed by quantifying wheel running activity using ClockLab under different lighting conditions, and then assessing their responses to light entrainment. Animals were exposed to regular 12-hour light and dark (LD) for 14 days, followed by constant darkness (DD) for 14 days, constant light (LL) for 19 days, before returning to regular 12 hour LD cycles for 23 days. Significantly, the results indicated strain-specific differences in circadian activity profiles under standard vivarium 12 hour LD cycles (baseline). Whereas COP rats demonstrated brief and high activity in response to light changes (i.e., off and on), F344 had a protracted activity pattern during darkness, suggesting that in F344 rats prevailing light conditions had a greater influence than changing conditions. This prediction was confirmed when lighting conditions were changed to DD, LL and back to LD cycles (Figure 5A). The animals were then shifted to DD to reveal their endogenous rhythm. The results demonstrated that after a period of adjustment, the activity profiles of the COP rats became more analogous to that of the F344 rats, showing activity between ZT12 and ZT24. In addition, the onset of running activity of COP was 1 hour earlier than that in F344 at lights off at 18:00 (ZT12) (Figure 5A). This suggested that the main difference in circadian rhythm between these strains was their response to light change. The ClockLab data further indicated that prolonged exposure to abnormal lighting altered the responsiveness of the COP rats to light entrainment. After being exposed to 14 days of DD followed by 19 days of LL, the COP rats no longer showed increased activity as a sign of sensitivity to lighting changes (i.e., on and off) in lighting. Instead, their overall response to a normal LD cycles more closely resembled the protracted activity seen in F344 rats, although COP rats were more accurately response to lights off or on than F344 (Figure 5A). These changes in diurnal behavior suggested that long periods of abnormal lighting might reprogram epigenetic regulation of the circadian clock in the COP strain.

ClockLab analysis results showed that the period of endogenous rhythm is significantly shorter and was sensitive to the changes in light to LL and back to LD in F344 rats, while the period was unaffected in COP rats. When rats were returned to regular LD cycles, it took three weeks to resynchronize the period of F344 rats to 24 hour LD cycles, compared to one week in COP rats (Figure 5B). Similary, the circadian phase of F344 also showed significant change in response to changing light conditions, but was unaffected in COP rats (Figure 5C). Together, these results indicated that COP rats were very sensitive to and responsed rapidly to changes in lighting without a significant change in circadian period and phase. By contrast, F344 rats are relatively insensitive and respond slowly to changes in lighting, indicating that the susceptible strain may harbor a genetic defect in genes controlling the input pathway to the circadian pacemaker or in the circadian clock itself.

**Figure 6:**
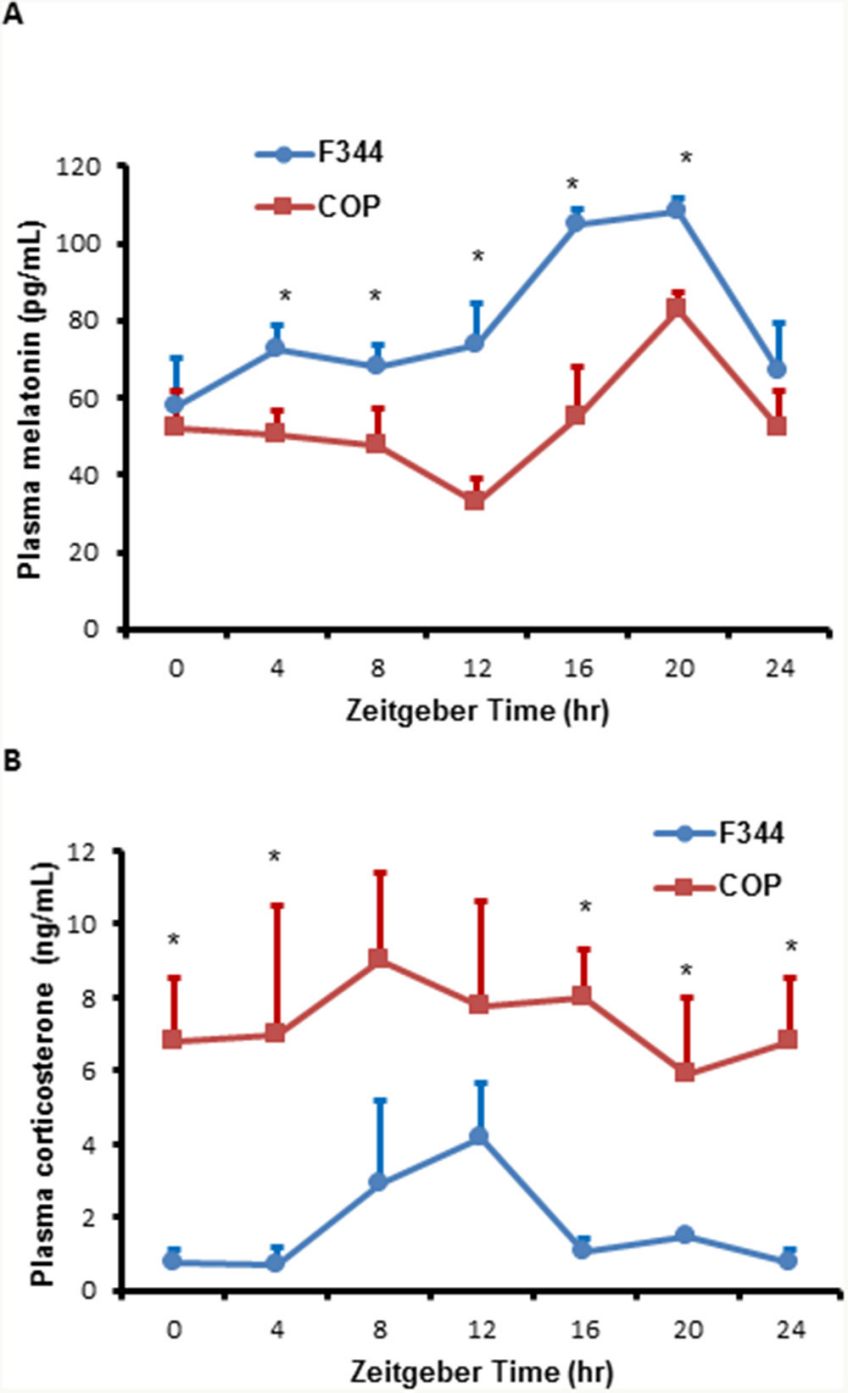
Comparison of plasma melatonin and corticosterone levels Three pubescent female COP (square) or F344 (circle) rats were sacrificed every 4 hours over a 24 hour period, beginning at 6 AM. **(A)** Plasma melatonin levels were determined using ELISA kit and plotted against Zeitgeber time (ZT). **(B)** Plasma corticosterone levels were determined using ELISA kit and plotted against Zeitgeber time (ZT). Values of mean ±SE (n=3 for each) are presented. * indicates statistical significance at p<0.05 between COP and F344 rat strains at the same time points.

In rodents, circadian release of corticosterone is an important mediator and modulator of circadian rhythm in peripheral cells. To assess the potential impact of strain specific differences in diurnal variation in hormone levels, we compared diurnal 24-hour profiles of plasma melatonin and corticosterone. F344 and COP rats showed similar 24-hour circadian profiles in plasma melatonin levels, although the overall levels were higher in F344 relative to COP rats (Figure 6A). These results suggested that the observed differences in locomotor activity did not significantly affect melatonin secretion. Moreover, plasma corticosterone levels had a significant circadian rhythm in F344 rats, with peak values at ZT12. Although the overall levels of corticosterone were higher in COP compared to F344 rats, levels of this stress hormone did not show a rhythmic pattern in COP rats (Figure 6B).

### Jet-lag abolished rhythmic expression of *Per2* and DDRR genes, and accelerated mammary tumorigenesis

**Figure 7:**
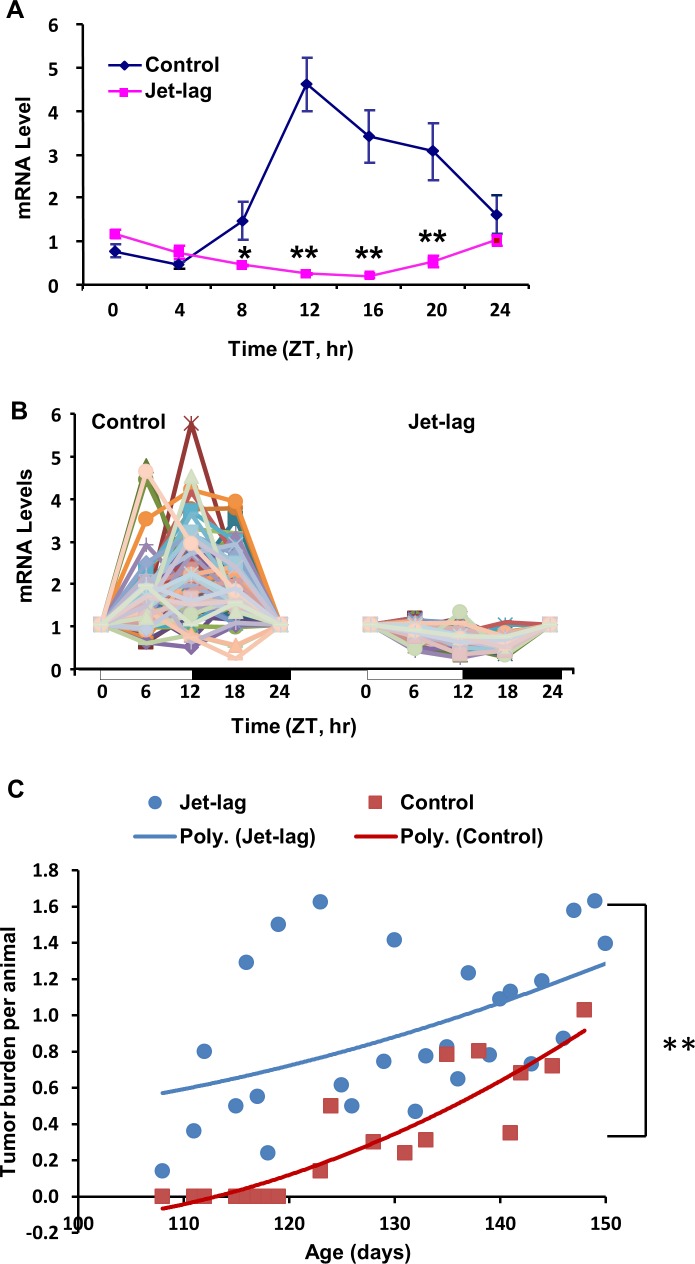
Circadian mRNA expression of *Per2* and DDRR genes, and mammary tumorigenesis in rodents COP Rats maintained on regular or jet-lagged LD cycles for 7 days were sacrificed every 4 **(A)** or 6 **(B)** hours over 24 hour, beginning at 6 AM, on the 2^nd^ day after returning to regular LD cycles. **(A)** RT-qPCR was employed for determination of *Per2* mRNA levels. *, p<0.05; **, p<0.01 (one way ANOVA, n=3). **(B)** RT-qPCR array was used for the determination of 84 DDRR genes mRNA levels. **(C)** Female C3(1)/Tag transgenic mice were maintained on jet-lagged or regular LD cycles beginning at 4-week age until termination at the indicated times. The average of total tumor burden per animal was analyzed as a function of tumor number and size measured. Control, n=29; Jet-lag, n=35. Regression analysis is presented as a polynomial trend line. ** indicates statistical significance at P<0.01.

To determine if altered light/dark cycles can disrupt circadian expression of CGs and DDRR genes in the resistant COP strain, we exposed female COP rats to a jet-lag protocol mimicking circadian disruption associated with day/night shift work or frequent cross-time-zone travel. Our results showed that jet-lag not only disrupted rhythmic expression of *Per2* (Figure 7A), but also reduced rhythmic expression of most DDRR genes (Figure 7B) in mammary glands of the resistant COP strain. Moreover, our studies on the effect of jet lag on mammary tumorigenesis in C3(1)/SV40 T-antigen [C3(1)/Tag] transgenic mice, further indicated that chronic jet lag (3-4 months) advanced mammary tumor onset by 16 days, increased tumor multiplicity by 50% at 140 days of age, and significantly increased tumor burden per animal (P<0.01) compared to regular LD cycles. The effects occurred at the early stage (100-150 days of age) of tumorigenesis in this mammary tumorigenesis model [60].

## DISCUSSION

Previous studies showed that *Per2* null mice have increased susceptibility to DNA damage and tumorigenesis [22, 23]. However, the circadian clocks in these mice are permanently fixed in either “on” or “off” state, precluding mechanistic studies of stressors and chemopreventive agents that alter carcinogenesis by modulating circadian gene expression. By contrast, our studies used rat strains with intact circadian clocks that have distinct patterns of circadian gene regulation at baseline and in response to endogenous and exogenous factors. Comparing these strains affords the opportunity to elucidate genetic and epigenetic mechanisms underlying circadian control of DDRR pathways and other networks involved in mammary carcinogenesis.

As reported by others, our previous studies demonstrated that while the F344 strain is highly sensitive to mammary carcinogenesis following exposure to the chemical carcinogen, NMU, the COP strain is highly resistant [46, 47]. In the present study, our results indicated that genetic background has profound influences on the regulation of circadian gene expression. In mammary glands of the COP rats, circadian expression of the core clock gene, *Per2*, was tightly coupled to the rhythmic expression of DDRR genes. Circadian expression of *Per2* and DDRR genes were sustained following exposure to NMU. In contrast, F344 rats exposed to carcinogen failed to reset *Per2* and DDRR gene expression, and even suppressed the rhythmic expression of many DDRR genes (e.g., DNA repair genes, *p53*, *p21*, and *Gadd45α*) [20]. Importantly, a chemopreventive regimen of dietary MSC restored circadian expression of clock (i.e., *Per2*) and DDRR genes towards the levels in mammary glands of resistant COP rats, reducing the incidence of mammary tumors by 63% in NMU-treated F344 [20, 46]. These results suggest that uncoupling of DDRR from circadian control reduces the ability of cells to resist the deleterious effects of DNA damage, and that this process can be reversed by MSC.

**Figure 8:**
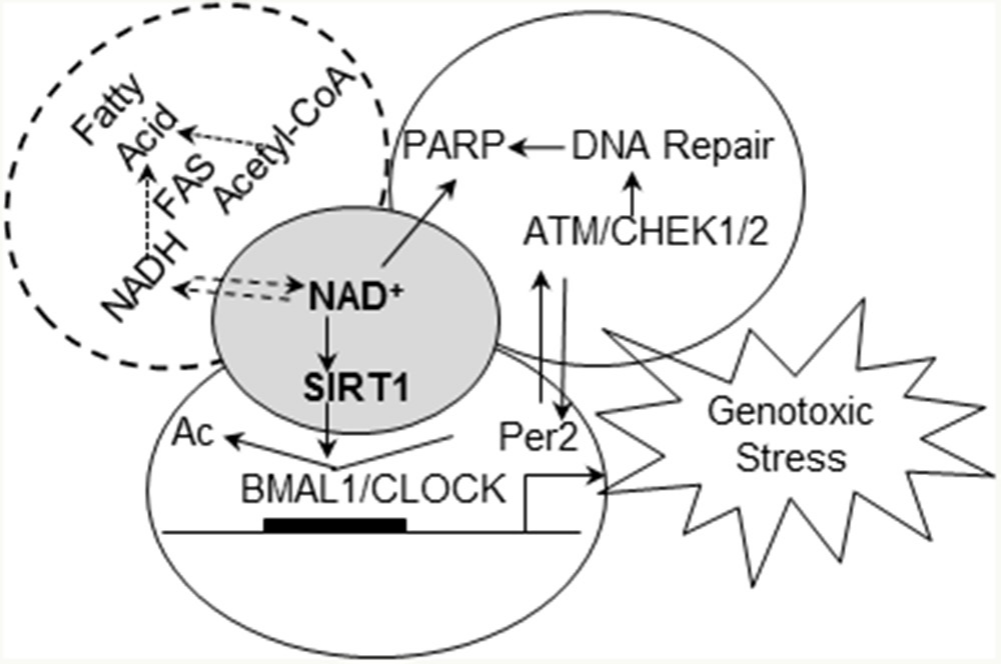
Working hypothesis of how circadian rhythm integrates signals from endogenous factors and genotoxic stresses to couple DDRR to circadian control NAD^+^-dependent SIRT1 deacetylase activity plays a central role in circadian regulation of DDRR (including PARP mediated DNA repair) at differential metabolic conditions, contributing to differential susceptibility to breast cancer.

Several mechanisms could contribute to reduced ability of F344 rats to respond to genotoxic stress. One possibility is that differences in their endogenous metabolic processes affect the capacity of the circadian clock to endure the consequences of genotoxic stress. Previous studies demonstrated strain differences in fatty acid synthesis, insulin resistance [61], and susceptibility to high-fat diet induced obesity [62]. Increases in each of these metabolic processes promote mammary tumorigenesis and are controlled by and/or affect circadian rhythm. Our observation that COP and F344 rat strains differ significantly in the rhythmic expression of *Rev-ErbAα*, a core circadian gene involved in adipogenesis, further links circadian altered control of cellular metabolism to carcinogenesis [60]. One possible mechanisms connecting these processes is changes in redox levels and/or cycling. Intracellular NAD^+^ levels are affected by redox cycling and cell metabolism, and can be depleted by poly (ADP-ribose) polymerase (PARP)-mediated DNA repair after genotoxic stress [63]. NAD^+^-dependent SIRT1 activity modulates the activity of the CLOCK:BMAL1 circadian transcription factor complex, linking cellular metabolism and redox cycling to a wide variety of cellular/molecular functions, including cellular circadian rhythm and DDRR [4, 64]. Therefore, we hypothesize that NAD^+^-dependent SIRT1 functions as the central coordinator/integrator of cellular circadian responses to endogenous and environmental conditions and stressors (Figure 8). Under normal conditions, circadian regulation ensures that cells have adequate reducing power and NAD^+^ stores before mounting a response to DNA damage. Depletion of intracellular NAD^+^ stores reduces NAD^+^-dependent SIRT1 activity, impairs circadian gene expression and uncouples DDRR from circadian control. Although arresting DNA repair prevents further depletion of NAD^+^ by PARP during excision repair, uncoupling DDRR from circadian control also creates a promutagenic state. Thus, strains with chronically reduced NAD^+^/NADH levels would be more susceptible to mutagenesis and carcinogenesis.

Consistent with the latter contention, our studies indicated that compared to the resistant COP strain, F344 rats have a reduced ability to sustain NAD^+^/NADH stores and to maintain SIRT1 activity after exposure to genotoxic stress. In F344 rats treated with NMU, reduction in circadian expression of *Per2* and CCGs occurred gradually over a 30-day period [57]. Since NMU has a half-life of ~30 minutes *in vivo*, these observations suggest that although abolition of circadian control was initiated by NMU, the epigenetic reprogramming probably occurs via an indirect mechanism. The dose of NMU (50 mg/kg) used in our studies induces significant genotoxic stress, and the resulting increase in DNA repair could cause acute depletion of NAD^+^ stores. The dose of NMU used also induces death of ~ 65% of the cells comprising the mammary tissue [65]. While this level of cell killing will induce inflammation and oxidative stress in both rat strains, NMU-treated COP rats were able to sustain SIRT1 activity and expression of genes involved in DDRR. However, F344 mammary glands are unable to maintain NAD^+^-dependent SIRT1 activity after NMU exposure, leading to further suppression of NAD^+^ biosynthesis and ablation of circadian expression of DDRR genes. The resulting promutagenic state no doubt contributes to mammary carcinogenesis, as evinced by the fact that restoration of circadian control in the F344 mammary gland by MSC dramatically reduced NMU-induced carcinogenesis [46]. Together these findings suggest that mammary glands of the F344 rats have reduced NAD^+^/NADH stores that are rapidly depleted by NMU exposure, leading to decreased SIRT1 activity in response to genotoxic stress. Consistent with our findings, reduction of NAD^+^ promoted breast cancer metastasis in rodent models [66], while enhancement of NAD^+^/NADH by NAD^+^ precursors (nicotinamide riboside) inhibited mammary tumor growth and metastasis, and increased survival [67]. The beneficial effects of exercise, calorie restriction, and diet polyphenols (i.e., resveratrol) on cancer prevention and life-span extension also appear to be mediated by NAD^+^/NADH and NAD^+^-dependent SIRT1 activity [68, 69]. Taken together, endogenous and environmental factors that decrease NAD^+^-dependent SIRT1 activity may therefore play a crucial role during the early stages of carcinogenesis by disrupting circadian control on DDRR (Figure 8).

An alternative explanation is that both central and peripheral circadian rhythm of F344 rats are inherently less robust than in the resistant COP strain. These differences could be reflected by differences in circadian levels of circulating stress hormones such as corticosterone [70, 71], since rat strains are known to have significant differences in their stress responses and regulation of their hypothalamic-pituitary-adrenal axis [72, 73]. We found that COP rats have a short, focused, and increased activity in response to changes of lighting, while F344 rats have a protracted period of activity during darkness. When light conditions were changed from LD to DD, LL and back to LD, COP rats rapidly synchronized their rhythm to LD cycles. By contrast, F344 were much slower to adjust their rhythm back to LD cycles. Despite significant difference in locomotor activity and gene expression in mammary glands between these two strains at regular LD cycles, their 24-hour melatonin profiles did not show signicant differences. While corticosterone levels were higher in COP rats, their diurnal levels were not affected by changing light conditions. These results indicated that central clock-controlled circadian fluctuations in circulating hormone levels are not likely to be associated with susceptibility to mammary carcinogenesis. However, differential sensitivity of the strains to light change was similar to with the sensitivity of *Per2* gene expression to genotoxic stress. Together, these findings suggest that genetic variations controlling intrinsic baseline circadian rhythm and their responses play a predominent role in differential susceptibility to carcinogenesis.

Our analysis of diurnal physical activity patterns further suggested that increased susceptibility to NMU-induced mammary carcinogenesis was associated with an abnormal circadian response to changing light conditions. COP rats were most active during the first hour after lights on and lights off, as seen in most prey species in the wild. By contrast, F344 rats displayed protracted activity after lights off. They also were slow in resetting their circadian rhythm after changing light condition from LL to LD. Disruption of normal LD cycles, such as constant light (LL) and jet-lagged LD cycles that mimic rotating shift work and frequent cross-time-zone travel, is known to promote carcinogenesis. Unlike carcinogen NMU, which works directly on clocks in peripheral cells [20], abnormal LD cycles disrupt the circadian rhythm of peripheral organs through central clock. We therefore asked whether exposure of COP rats to constant light or jet-lagged LD cycles can disrupt circadian controlled behavior, and expression of the major clock genes and DDRR genes. Our studies demonstrated that prolonged exposure to constant light dramatically altered the response of COP rats to changing light conditions. After changing back to normal LD cycles, they no longer showed the normal pattern of response to changes in lighting. Moreover, jet-lagged LD cycles not only disrupted circadian gene expression, but also ablated circadian expression of DDRR genes in COP rats. The expression patterns of these genes in the COP rats maintained in jet-lagged LD cycles closely resembled those in the NMU-exposed F344 rat. Studies to determine if these changes in circadian control of DDRR also increase the susceptibility of COP rats to mammary carcinogenesis are in progress. As an alternative approach, we demonstrated that chronic exposure to jet-lagged LD cycles advanced mammary tumor onset and accelerated tumor growth in breast cancer-prone transgenic mice. Similar study also showed that weekly LD inversions decreased tumor suppression, resulting in increase of breast cancer development in mammary gland conditional p53 mutant mice [74]. Together with previous findings [11, 12], these results further suggest that circadian disruption by abnormal lighting conditions promotes spontaneous and carcinogen-induced carcinogenesis in rodents, regardless of genetic background. It would be interesting to determine whether the increase in tumorigenesis by jet-lag can be mitigated by chemopreventive agents (e.g., MSC) in these animal models.

In the present study, we demonstrated that genetic variation affecting circadian responses to abnormal lighting conditions and genotoxic stress affects susceptibility to carcinogenesis. Enhancing and recoupling circadian control to DDRR may therefore be a productive intervention strategy for individuals who are at elevated risk of breast cancer as a result of genetic predisposition and/or environmental/occupational exposures.

## MATERIALS AND METHODS

### Ethical statement

All animal experiments were performed in AAALAC accredited facilities at Rutgers, The State University of New Jersey using protocols approved by our Institutional Animal Care and Use Committee (IACUC).

### Rat treatment and sample collection

Pubescent female Fisher 344 (F344) and Copenhagen (COP) rats (Charles River) were maintained on a standard research diet (AIN-76A). Animals were housed under controlled conditions with a 12 hour LD cycle. Zeitgeber Time 0 (ZT 0) was set at 6 AM (light on); ZT12 was set at 6 PM (light off).

NMU (Sigma) was dissolved in acidified saline (pH 5) to a concentration of 10 mg/ml immediately before injection. For time course studies, female F344 and COP rats (aged 55±2 days, 155-175 g) treated with NMU (at 2-4 pm) by a single intra-peritoneal (*i.p*.) injection (50 mg/kg body weight) were randomized to 3 groups (n=21 per group per strain). Groups 1, 2, and 3 were sacrificed at days 0, 2, and 30 post-exposure, respectively. Additionally, one group (n=21) of F344 rats treated with NMU were maintained on MSC-enriched diet (3 ppm Se in MSC form) for 30 days, beginning on the day of exposure to NMU. For jet-lag studies, female COP rats were randomized into two groups (n= 21 for each), exposed to jet-lagged or regular 12 hour LD cycles for 1 week, and sacrificed at day 2 after returning to regular LD cycles. The jet-lagged LD cycle is initiated by advancing the light onset by 12 hours and maintained on switched 12 hour LD cycle for 7 days. On the day of shift, the light period is lengthened to 24 hours; on the day of shift back to regular LD cycle, the light period is shortened to 0 hour (i.e., dark period is lengthened to 24 hours). Three rats per group were sacrificed by CO_2_ asphyxiation every 4 (or 6 hours for RT-qPCR array) hours over a 24-hour period, beginning at 6 AM (ZT0). Blood samples were collected into heparinized-BD Vacutainers by cardiac punctures after CO2 asphyxiation. Plasma samples were separated by sequential centrifuge (at 1300 rcf for 10 min first and then at 2400 rcf for 15 min) and stored at -80°C. Mammary glands at each side of individual rats were carefully dissected, combined into a pool of left or right mammary gland tissue sample. Tissue samples were snap-frozen on dry ice, and then stored at -80°C.

### Determination of plasma corticosterone levels in rats

Corticosterone levels were quantified with a competitive immunoassay using Corticosterone ELISA kit (Enzo Lift Science) and SpectraMax V5 microplate reader (Molecular Devices) according to the manufacturer's instruction.

### Determination of plasma melatonin levels in rats

Melatonin levels were determined using Melatonin ELISA Kit (GenWay Biotech) and SpectraMax V5 microplate reader (Molecular Devices) according to the manufacturer's instruction.

### Determination of locomotor activity in rats

Five-week-old rats (F344 and COP, 3 for each strain) were placed in standard rat cage equipped with infrared sensors to detect free wheel running activity using ClockLab (Actimetrics). After 2-week of running at regular 12 hour LD cycles, animals were maintained in DD for 2 weeks, LL for 19 days, and then regular LD cycles for 23 days. Data collected from individual rats were analyzed with ClockLab Analysis software (Actimetrics). Actograms, periodograms, and activity profiles were generated to determine period, amplitude, and phase. These parameters were compared between F344 and COP rat strains maintained under different lighting conditions.

### Determination of the effect of jet-lag on mammary tumorigenesis

Heterozygous female [C3(1)/Tag] mice (Jackson Lab) were selected and maintained on AIN76A diet and regular LD cycles. After weanling at 4 weeks of age, animals were assigned to Group 1 (n=29) - continue to be maintained in the regular LD cycles and Group 2 (n=35) – maintained on jet-lag, which mimics rotating shift work with 8 hour -advanced or delayed light onset very 3 days. On the day of shift, the time of lights-on is advanced or delayed by 8 hours, so that the dark period is shortened to 4 hours or lengthened to 20 hours. Tumor number and size were measured using a caliper twice a week. Tumor volume was calculated with a standard method (V=(W^2xL)/2) [75]. Tumor onset, multiplicity, and average total tumor burden per animal were compared.

### Quantitative real-time RT-PCR

Total RNA was extracted from a small piece (~50 mg) of frozen tissue from right-side mammary glands of rats. *Per2* mRNA expression levels were determined using real-time quantitative RT-PCR as described previously [20].

### Quantitative real-time RT-PCR array

DDRR genes’ mRNA expression levels were determined with RT-qPCR array kit, Rat DNA Damage Signaling PCR Array (Qiagen, Supplementary Table 1), according to the manufacturer's instruction. Pooled mammary gland samples were obtained from 3 rats (50 mg for each) per time point per group, and total RNA samples were extracted and used in RT-qPCR array. Results were analyzed with RT^2^ Profiler PCR Array Data Analysis version 3.5 (Qiagen).

### Western blot

Mammary gland samples from 3 rats (50 mg for each) were pooled per time point per group. Total protein was extracted using RIPA lysis buffer (50 mM Tris-HCl, pH 7.5, 150 mM NaCl, 1 mM EDTA, 0.5% Sodium-deoxycholate, 0.1% SDS) supplemented with a cocktail of protease inhibitor (1:100), phosphotase inhibitor I (1:100), and phosphotase inhibitor II (1:100) (Sigma-Aldrich). Forty μg of protein sample was separated on 7.5% SDS-polyacrylamide gel and transferred to nitrocellulose membrane. After blocking, the membrane was incubated with primary antibody, anti-rat Mutyh (Novus Biological) or anti-rat β-Actin (Sigma) overnight at 4°C, followed by incubation with IR Dye 800 CW anti-goat or anti-rabbit IgG (LI-COR). Targeted protein signals with fluorescence were detected using Odyssey Infrared Imaging System (LI-COR). β-Actin was used as an internal normalization control.

### SIRT1 activity assay

Total protein extracts were prepared from a small piece (~50 mg) of tissue from right-side mammary gland of individual rats sacrificed at ZT12 on day 30 post-exposure, or from cultured cells with RIPA buffer. SIRT1 deacetylase activity was determined with SIRT1 Fluorimetric Drug Discovery Kits (Enzo Life Sciences). Briefly, initial deacetylation rates of SIRT1 were determined at 1 unit human recombinant SIRT1 enzyme, 25 μM deacetylase substrate, and 25 μM NAD^+^ (37°C) in the absence (control) or presence of 10 μl of extracted protein. Fluorescence signal was measured with a microplate reader at 360 nm excitation and 460 nm emission wavelengths. Standard curve was produced with serially diluted-deacetylation standard. Activity was normalized to protein concentration and expressed as deacetylated product (μmol)/protein (μg). Three independent samples per group were analyzed in triplicate.

### Statistical analyses

Animal number *N* = 3 was used in rat *in vivo* studies. Intergroup differences were evaluated using one-way ANOVA, followed by Tukey's *post-hoc* test using GraphPad InStat 3 (GraphPad Software). We used *alpha* = 0.05 or 0.01 as the level of significance for hypothesis testing. Analysis of Covariance (ANCOVA) was used to determine the significance of jet-lag vs control on the overall level of mouse tumor burden using a SAS Software for Windows, Version 9.4 (SAS Institute).

## SUPPLEMENTARY MATERIALS FIGURES AND TABLES





## Abbreviations

CG: circadian gene
CCG: circadian controlled gene
Per2: Period 2
PARP: poly (ADP-ribose) polymerase
DDRR: DNA damage response and repair
ZT: Zeitgeber time
LD: light and dark
DD: constant darkness
LL: constant light
MSC: methylselenocysteine
NMU: N-Nitroso-N-methylurea
F344: Fisher 344
COP: Copenhagen
C3(1)/Tag: C3(1)/SV40 T-antigen
