# Supplementary material for: Uncoupling genotoxic stress responses from circadian control increases susceptibility to mammary carcinogenesis
Source: Oncotarget. 2017 Feb 24;8(20):32752–68. doi: 10.18632/oncotarget.15678 (PMC5464825; doi:10.18632/oncotarget.15678)
Supplement: Supplementary file 1 [file oncotarget-08-32752-s001.pdf]

Uncoupling genotoxic stress responses from circadian control increases susceptibility to mammary carcinogenesis

SUPPLEMENTARY TABLE

Supplementary Table 1: Gene List in Rat DNA Damage Signaling Pathway RT-qPCR Array (Qiagen, Catalog # PARN-029)

See Supplementary File 1
