# Supplementary material for: Uncoupling genotoxic stress responses from circadian control increases susceptibility to mammary carcinogenesis
Source: Oncotarget. 2017 Feb 24;8(20):32752–68. doi: 10.18632/oncotarget.15678 (PMC5464825; doi:10.18632/oncotarget.15678)
Supplement: Supplementary file 2 [file oncotarget-08-32752-s002.docx]

Supplemental Table 1. Gene List in Rat DNA Damage Signaling Pathway RT-qPCR Array (Qiagen, Catalog # PARN-029).

| **Refseq** | **Symbol** | **Description** |
| --- | --- | --- |
| NM_024148 | Apex1 | APEX nuclease (multifunctional DNA repair enzyme) 1 |
| XM_236275 | Atm | Ataxia telangiectasia mutated homolog (human) |
| XM_217570 | Atrx | Alpha thalassemia/mental retardation syndrome X-linked (RAD54 homolog, S. cerevisiae) |
| NM_012514 | Brca1 | Breast cancer 1 |
| NM_031542 | Brca2 | Breast cancer 2 |
| NM_080400 | Chek1 | CHK1 checkpoint homolog (S. pombe) |
| NM_133405 | Cry2 | Cryptochrome 2 (photolyase-like) |
| NM_031583 | Smc3 | Structural maintenance of chromosomes 3 |
| XM_214730 | Dclre1a | DNA cross-link repair 1A, PSO2 homolog (S. cerevisiae) |
| XM_214833 | Ercc1 | Excision repair cross-complementing rodent repair deficiency, complementation group 1 |
| XM_218424 | Ercc2 | Excision repair cross-complementing rodent repair deficiency, complementation group 2 |
| XM_222932 | Exo1 | Exonuclease 1 |
| NM_012557 | Fancc | Fanconi anemia, complementation group C |
| NM_053430 | Fen1 | Flap structure-specific endonuclease 1 |
| NM_024127 | Gadd45a | Growth arrest and DNA-damage-inducible, alpha |
| XM_341863 | Gtf2h1 | General transcription factor IIH, polypeptide 1 |
| XM_215466 | Gtf2h2 | General transcription factor IIH, polypeptide 2 |
| NM_199388 | Dpagt1 | Dolichyl-phosphate (UDP-N-acetylglucosamine) N-acetylglucosaminephosphotransferase 1 (GlcNAc-1-P transferase) |
| NM_030855 | Lig1 | Ligase I, DNA, ATP-dependent |
| XM_343671 | Chaf1a | Chromatin assembly factor 1, subunit A (p150) |
| XM_573658 | Hus1 | HUS1 checkpoint homolog (S. pombe) |
| XM_001059437 | Mbd4 | Methyl-CpG binding domain protein 4 |
| XM_001061612 | Rbbp4 | Retinoblastoma binding protein 4 |
| XM_001076851 | Fancg | Fanconi anemia, complementation group G |
| NM_001025701 | Rad21 | RAD21 homolog (S. pombe) |
| NM_001009535 | Pms1 | PMS1 postmeiotic segregation increased 1 (S. cerevisiae) |
| NM_012861 | Mgmt | O-6-methylguanine-DNA methyltransferase |
| NM_031051 | Mif | Macrophage migration inhibitory factor |
| NM_031053 | Mlh1 | MutL homolog 1 (E. coli) |
| XM_234420 | Mlh3 | MutL homolog 3 (E. coli) |
| NM_012601 | Mpg | N-methylpurine-DNA glycosylase |
| NM_022279 | Mre11a | MRE11 meiotic recombination 11 homolog A (S. cerevisiae) |
| NM_031058 | Msh2 | MutS homolog 2 (E. coli) |
| XM_001065837 | Msh3 | MutS homolog 3 (E. coli) |
| NM_133316 | Mutyh | MutY homolog (E. coli) |
| XM_213228 | Nthl1 | Nth (endonuclease III)-like 1 (E.coli) |
| NM_030870 | Ogg1 | 8-oxoguanine DNA glycosylase |
| NM_013063 | Parp1 | Poly (ADP-ribose) polymerase 1 |
| XM_214157 | Parp2 | Poly (ADP-ribose) polymerase 2 |
| XM_213712 | Pms2 | PMS2 postmeiotic segregation increased 2 (S. cerevisiae) |
| NM_021662 | Pold1 | Polymerase (DNA directed), delta 1, catalytic subunit |
| NM_001024750 | Pold3 | Polymerase (DNA-directed), delta 3, accessory subunit |
| XM_222255 | Pole | Polymerase (DNA directed), epsilon |
| XM_236934 | Polh | Polymerase (DNA directed), eta |
| XM_225844 | Poli | Polymerase (DNA directed), iota |
| XM_342178 | Polk | Polymerase (DNA directed) kappa |
| XM_341020 | Prkdc | Protein kinase, DNA activated, catalytic polypeptide |
| NM_022391 | Pttg1 | Pituitary tumor-transforming 1 |
| XM_215497 | Rad1 | RAD1 homolog (S. pombe) |
| NM_001024778 | Rad17 | RAD17 homolog (S. pombe) |
| XM_342734 | Rad18 | RAD18 homolog (S. cerevisiae) |
| NM_001013190 | Rad23a | RAD23 homolog A (S. cerevisiae) |
| NM_022246 | Rad50 | RAD50 homolog (S. cerevisiae) |
| NM_001109204 | Rad51 | RAD51 homolog (RecA homolog, E. coli) (S. cerevisiae) |
| XM_576058 | Rad51l1 | RAD51-like 1 (S. cerevisiae) |
| XM_216230 | Rad52 | RAD52 homolog (S. cerevisiae) |
| XM_219684 | Rad9 | RAD9 homolog (S. pombe) |
| NM_001030042 | Rad9b | RAD9 homolog B (S. cerevisiae) |
| XM_215201 | Rbm4 | RNA binding motif protein 4 |
| XM_237071 | Rev1 | REV1 homolog (S. cerevisiae) |
| NM_001034936 | Mare | Alpha globin regulatory element containing gene |
| XM_001081158 | Rad51c | Rad51 homolog c (S. cerevisiae) |
| XM_575317 | Xrcc2 | X-ray repair complementing defective repair in Chinese hamster cells 2 |
| XM_224282 | Pinx1 | PIN2-interacting protein 1 |
| NM_019349 | Slk | STE20-like kinase (yeast) |
| NM_031683 | Smc1a | Structural maintenance of chromosomes 1A |
| NM_022711 | Srd5a2 | Steroid-5-alpha-reductase, alpha polypeptide 2 (3-oxo-5 alpha-steroid delta 4-dehydrogenase alpha 2) |
| NM_001009672 | Sumo1 | SMT3 suppressor of mif two 3 homolog 1 (S. cerevisiae) |
| NM_053729 | Tdg | Thymine-DNA glycosylase |
| NM_001012464 | Terf1 | Telomeric repeat binding factor (NIMA-interacting) 1 |
| XM_242032 | Tlk1 | Tousled-like kinase 1 |
| NM_017056 | Tnp1 | Transition protein 1 |
| NM_030989 | Tp53 | Tumor protein p53 |
| NM_001024989 | Trex1 | Three prime repair exonuclease 1 |
| NM_022638 | Trpc2 | Transient receptor potential cation channel, subfamily C, member 2 |
| NM_001013933 | Ube2a | Ubiquitin-conjugating enzyme E2A, RAD6 homolog (S. cerevisiae) |
| NM_001013124 | Ung | Uracil-DNA glycosylase |
| XM_214361 | Wrn | Werner syndrome |
| NM_172332 | Wrnip1 | Werner helicase interacting protein 1 |
| XM_216403 | Xpa | Xeroderma pigmentosum, complementation group A |
| XM_232194 | Xpc | Xeroderma pigmentosum, complementation group C |
| NM_053435 | Xrcc1 | X-ray repair complementing defective repair in Chinese hamster cells 1 |
| NM_139080 | Xrcc6 | X-ray repair complementing defective repair in Chinese hamster cells 6 |
| XM_342535 | Xrn2 | 5'-3' exoribonuclease 2 |
| NM_001007604 | Rplp1 | Ribosomal protein, large, P1 |
| NM_012583 | Hprt1 | Hypoxanthine phosphoribosyltransferase 1 |
| NM_173340 | Rpl13a | Ribosomal protein L13A |
| NM_017025 | Ldha | Lactate dehydrogenase A |
| NM_031144 | Actb | Actin, beta |
| U26919 | RGDC | Rat Genomic DNA Contamination |
| SA_00104 | RTC | Reverse Transcription Control |
| SA_00104 | RTC | Reverse Transcription Control |
| SA_00104 | RTC | Reverse Transcription Control |
| SA_00103 | PPC | Positive PCR Control |
| SA_00103 | PPC | Positive PCR Control |
| SA_00103 | PPC | Positive PCR Control |
